# Combining H-Adaptivity with the Element Splitting Method for Crack Simulation in Large Structures

**DOI:** 10.3390/ma15010240

**Published:** 2021-12-29

**Authors:** Shi Song, Moritz Braun, Bjarne Wiegard, Hauke Herrnring, Sören Ehlers

**Affiliations:** Institute for Ship Structural Design and Analysis, Am Schwarzenberg Campus 4 c, Hamburg University of Technology, 21073 Hamburg, Germany; moritz.br@tuhh.de (M.B.); bjarne.wiegard@tuhh.de (B.W.); herrnring@tuhh.de (H.H.); ehlers@tuhh.de (S.E.)

**Keywords:** finite element method, mesh strategy, linear elastic fracture mechanics, mesh refinement, fracture mechanics, numerical crack, h-AES method, interelement method, edge separation, crack propagation

## Abstract

H-adaptivity is an effective tool to introduce local mesh refinement in the FEM-based numerical simulation of crack propagation. The implementation of h-adaptivity could benefit the numerical simulation of fatigue or accidental load scenarios involving large structures, such as ship hulls. Meanwhile, in engineering applications, the element deletion method is frequently used to represent cracks. However, the element deletion method has some drawbacks, such as strong mesh dependency and loss of mass or energy. In order to mitigate this problem, the element splitting method could be applied. In this study, a numerical method called ‘h-adaptive element splitting’ (h-AES) is introduced. The h-AES method is applied in FEM programs by combining h-adaptivity with the element splitting method. Two examples using the h-AES method to simulate cracks in large structures under linear-elastic fracture mechanics scenario are presented. The numerical results are verified against analytical solutions. Based on the examples, the h-AES method is proven to be able to introduce mesh refinement in large-scale numerical models that mostly consist of structured coarse meshes, which is also beneficial to the reduction of computational resources. By employing the h-AES method, very small cracks are well represented in large structures without any deletions of elements.

## 1. Introduction

The finite element method (FEM) is an effective tool for the simulation of static or cyclic crack propagation. In FEM-based simulations of crack propagation in large structures using shell elements, such as ship hulls, coarse meshes are often employed in consideration of computational cost [1,2]. However, the dimensions of cracks are relatively small in large structures. If a higher accuracy or more local details are needed in the numerical model, it is desirable to introduce local mesh refinement, which requires less computational cost than applying a fine mesh to the entire model [3,4]. In order to introduce local mesh refinement, the h-adaptivity could be applied. The h-adaptivity has been proven to be an effective tool to enhance local accuracy while keeping computational costs low, as can be seen in several research works on the simulation of cracks [5,6,7,8].

For engineering questions using shell elements, the element deletion method is often used to represent cracks. The element deletion method is easy to implement, and its accuracy can be enhanced in many ways—e.g., by using higher mesh density [9] or by adopting suitable material models [1,10,11,12,13]. As a result, the element deletion method is frequently applied in accidental load scenarios involving large structures, e.g., during ship collision and grounding [14,15,16]. However, the deletion of elements will bring some drawbacks. One of the drawbacks is the loss of mass or energy due to element deletion [17,18]. The other is the strong mesh dependency along the crack path, causing differences between the numerical and experimental results [19,20,21].

Apart from the element deletion method, there are other FEM-based crack representation methods—such as the extended finite element method (XFEM), the remeshing technique, the edge separation method, and the element splitting method. In the algorithm of the XFEM, the cracks are modelled by introducing functions that represent discontinuity in elements with fracture [22,23,24,25]. Compared with the element deletion method, crack paths based on the XFEM method could be less mesh-dependent [20,24,26,27,28], and the conservation of mass could be preserved [28,29]. Research combining XFEM with local mesh refinement were also proved to be effective [4,30]. Remeshing techniques are usually applied to rebuild local meshes around the crack tip [31,32,33,34,35], leading to very precise results regarding the crack initiation and propagation. However, the mesh rebuild process causes additional computational cost.

The edge separation method is also known as an interelement method [20]. In the edge separation method, the crack paths always coincide with the existing element edges. In order to separate the elements by their edges, the Xu–Needleman method [36] or the Camacho and Ortiz method [37] could be applied. When applied to shell elements, the edge separation method is often used together with cohesive methods [38,39,40] or the VCCT method [41]. For cracks induced by cyclic loading, compared to classical theory of linear elastic fracture mechanics (LEFM), this method allows for an easy implementation of uncertainty in fatigue crack initiation and propagation characteristics by means of random fields [42]. However, in the edge separation method, the crack path is strongly mesh-dependent, since it has to coincide with the element edges [43]. Some meshing techniques introduce more element edges to enhance the accuracy of a crack path, such as the 4k mesh technique [44,45,46], which has also been proven to be effective when combined with adaptive mesh refinement [45].

**Figure 1:**
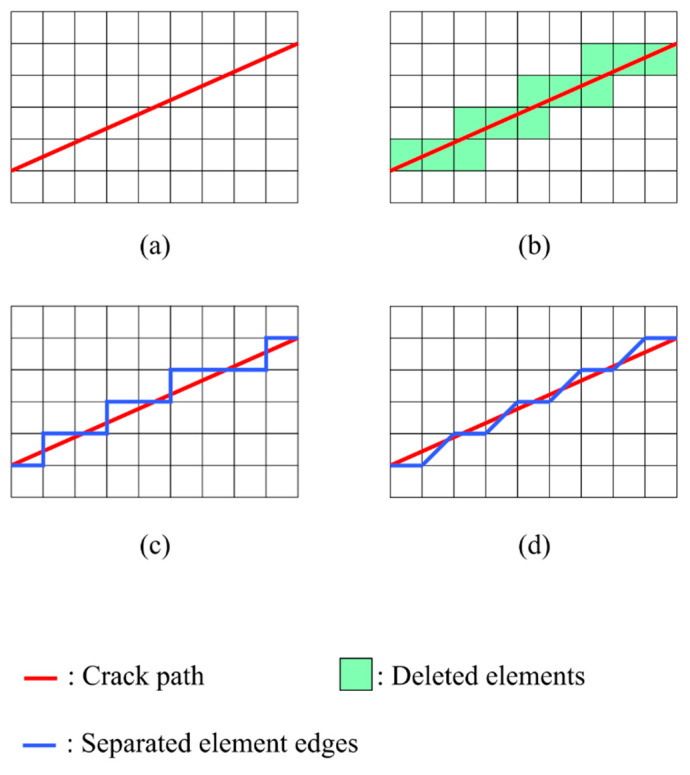
(**a**) The original mesh and the crack path; (**b**) Crack representation using the element deletion method; (**c**) Crack representation using the edge separation method; (**d**) Crack representation using the element splitting method.

In order to reduce the mesh dependency in the edge separation method, the element splitting method could be applied. In the element splitting method, a crack path still has to coincide with the element edges [47], just like in the edge separation method. However, the existing elements can be split up to create more element edges. Thus, cracks are considered to be able to propagate within the original elements. As a result, the mesh dependency, though still existent, could be reduced [47,48,49,50]. The element splitting method could be applied to hexagon elements [47,48,51], quadrilateral elements [49], triangular elements [52], or 4k mesh-based triangular and quadrilateral elements [50]. Figure 1 shows an example of the different crack representations resulting from element deletion, edge separation, and the element splitting method. More details about the element splitting method will be introduced later in this paper. However, one of the research articles above combined the element splitting method with h-adaptivity for the representation of cracks in large structures.

In this study, a combination of the h-adaptivity and the element splitting method, which is called the ‘h-adaptive element splitting’ (h-AES) method, is introduced. The aim of the h-AES method is to provide an optional alternative to current methods of simulating small cracks in large structures with a structured coarse global mesh. Compared with unstructured meshes, structured meshes can be generated and refined easier [47], which is the reason for applying structured meshes in this study. In Section 2, the basic concept and methodology of the h-AES method is introduced. Before applying it to more complex models, a verification against analytical solution is needed as a first step. Thus, in Section 3, two examples of numerical implementation using the h-AES method are presented, in which the numerical results are compared with analytical results based on LEFM. A further discussion of the h-AES method can be found in Section 4.

## 2. The H-Adaptive Element Splitting Method

### 2.1. Mesh Refinement Using H-Adaptivity

**Figure 2:**
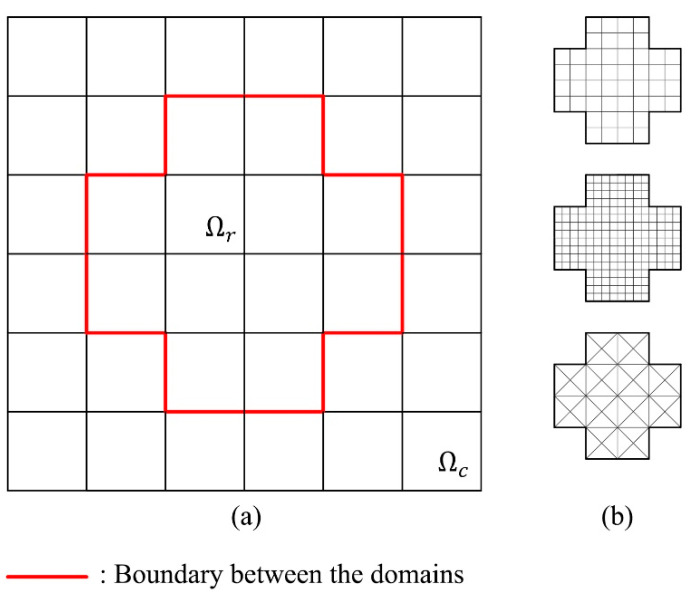
(**a**) Original coarse mesh with a region to be refined; (**b**) Optional mesh refinement of $\Omega_{r}$.

**Figure 3:**
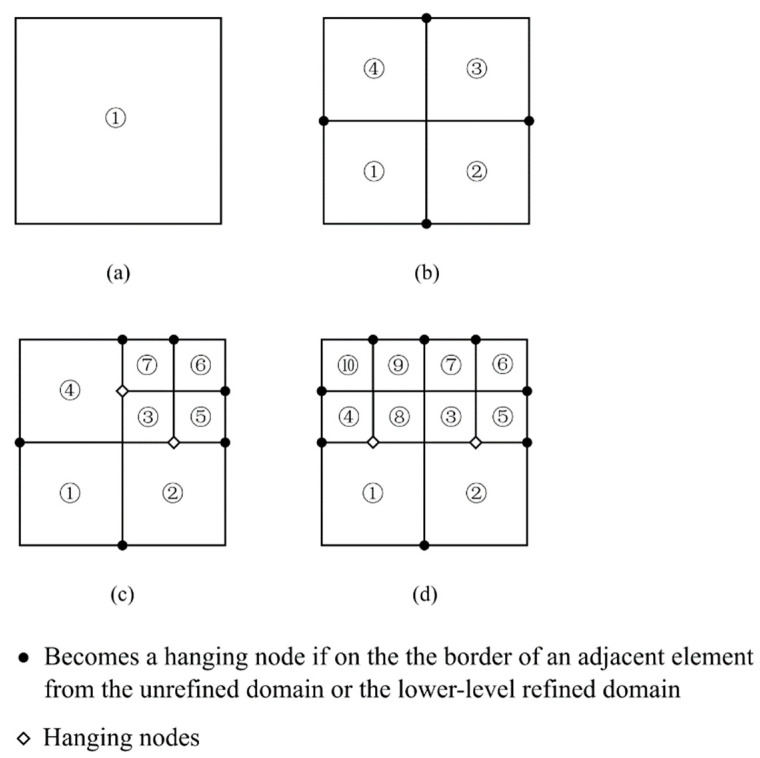
(**a**) A parent element before refinement; (**b**) Four sibling elements replacing the parent element; (**c**) One sibling element becomes a parent element for four sibling elements on the next level; (**d**) Two sibling elements become parent elements for eight sibling elements on the next level.

In the h-AES method, when local mesh refinement is introduced, the local mesh is divided into two domains: the domain of refined mesh $\Omega_{r}$ and the domain of the original coarse mesh $\Omega_{c}$; see Figure 2. The mesh refinement is introduced by dividing the ‘parent elements’ into ‘sibling elements’—a process that is also known ‘fission’ [5]; see Figure 3.

**Figure 4:**
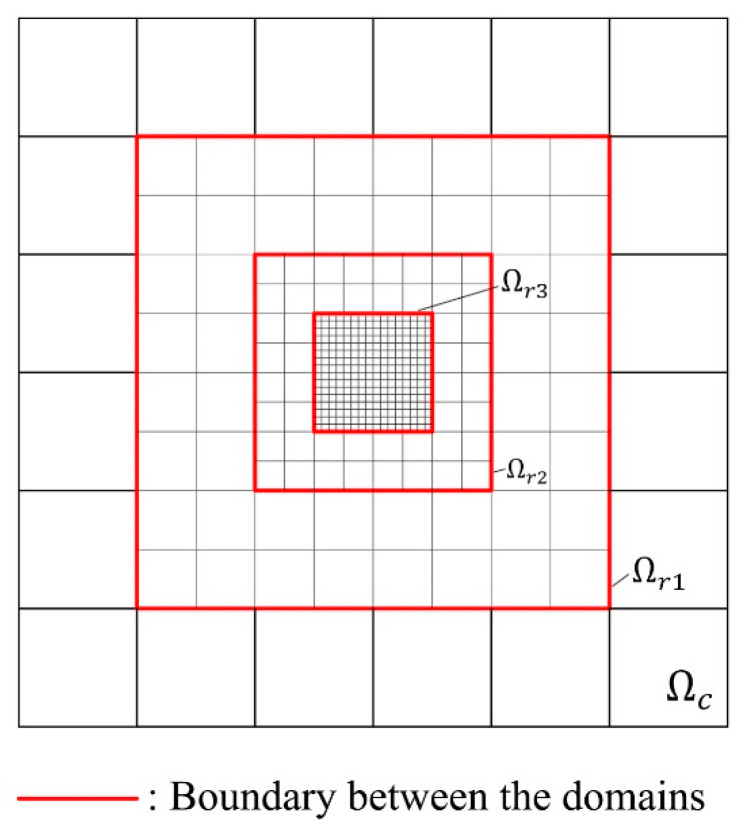
Multiple levels of mesh refinement, where the thinner lines represent the new element edges after refinement. $\Omega_{r1}$, $\Omega_{r2}$, and $\Omega_{r3}$ refer to the domain of first, second, and third level of refinement, respectively.

If a smaller mesh size is needed, the parent elements can be divided into more sibling elements; see Figure 2b. Additionally, further refinement could be introduced on the already refined meshes; see Figure 3c,d and Figure 4. The domains of the further refined mesh belong to different refinement levels. If more than one level of refinement is introduced, the first refined domain $\Omega_{r}$ is defined as $\Omega_{r1}$ instead, and the further refinement levels are defined as $\Omega_{r2}$ and so on; see Figure 4.

**Figure 5:**
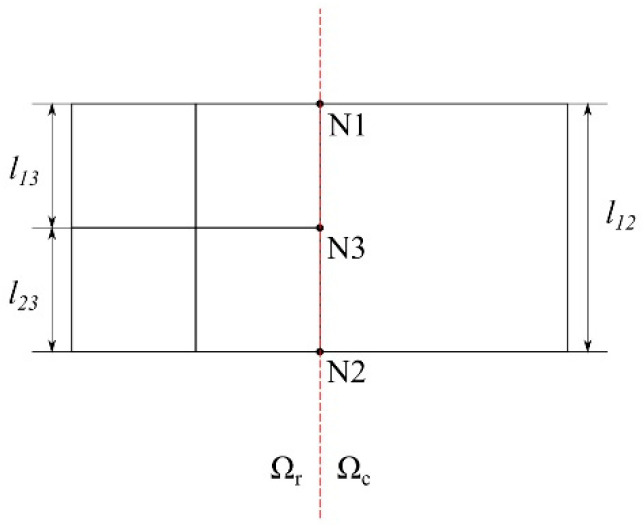
An example of the boundary nodes between two domains of mesh refinement, where N3 is a hanging node.

During the fission process, a newly generated node will become a hanging node if it is on the edge of a neighboring element from the unrefined domain or the lower-level refined domain; see Figure 3. If a hanging node is not on the crack path, an additional function is needed to keep the consistency between the domains. In the h-adaptivity, linear boundary conditions [6] are applied to the hanging nodes; see Figure 5. Considering the node relationship in Figure 5, the linear boundary condition could be expressed as:

(1) $$u_{3}=\frac{l_{23}}{l_{12}}u_{1}+\frac{l_{13}}{l_{12}}u_{2}$$

where $u_{1}$, $u_{2}$, and $u_{3}$ are the displacement of N1, N2, and N3, respectively.

### 2.2. Representation of Cracks

**Figure 6:**
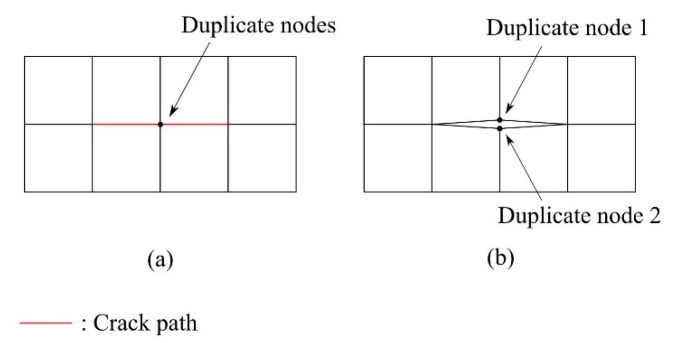
(**a**) Representation of a crack by introducing duplicate nodes on the crack path; (**b**) Crack-representation by separating the relevant edges of the elements in the mesh.

As is discussed before, the element splitting method is identical to the edge separation method if no element splitting happens. The edge separation algorithm used in the h-AES method is based on a method introduced by Camacho and Ortiz [37], where cracks are modelled by adding duplicate nodes along the crack paths; see Figure 6.

**Figure 7:**
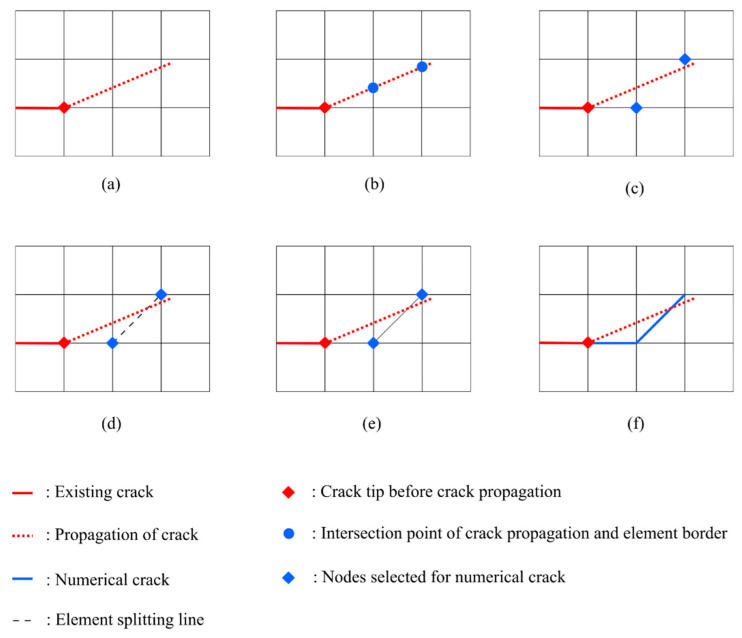
(**a**) Position of the crack and the crack propagation; (**b**) Intersection points of crack propagation and element edge are marked on the mesh; (**c**) Nodes with shortest distance to the intersection points are selected for the numerical crack; (**d**) One of the elements is split for the numerical crack; (**e**) Mesh after element splitting; (**f**) Numerical crack is represented by separating relevant element edges.

The element splitting process in the h-AES method only applies to quadrilateral elements. Figure 7 shows the procedure of modelling crack propagation using the element splitting method, in which the direction and length of crack propagation are known. At the beginning, the program will find the intersection point of the element edges and the propagation of the crack. After that, the nodes with the shortest distance to the intersection points are selected. The path of the numerical crack is determined by connecting the selected nodes. If any pair of neighboring nodes along the numerical crack are not connected by existing element edges (see Figure 7d), the element will be split along its diagonal line. The split element is replaced by two triangular elements; see Figure 7e. In addition, if the end position of the crack propagation does not coincide with any nodes, the program will find a node whose distance from the propagation end is less than $l_{e}$, where $l_{e}$ is the length of the element edge. The node that meets this condition will be included in the numerical crack as well. In the h-AES method, although the numerical crack is still mesh-dependent, the implementation of element splitting could provide more flexible crack paths than the normal edge separation method. Moreover, in structured meshes consisting of triangular and quadrilateral elements, the accuracy of a crack path can be enhanced by adapting smaller element sizes from mesh refinement [46].

## 3. Numerical Implementation in LEFM Using the H-AES Method

In this section, two numerical examples using the h-AES method are exemplarily presented for verification. Their numerical results are verified against analytical solutions based on LEFM theory.

In order to adapt the h-AES method for FEM calculation, a FEM-based MATLAB program was developed, including the modules of pre-processing, calculation, and post-processing. In this study, since the two examples are both 2D models, 4-nodes quadrilateral elements and 3-nodes triangular elements are used. The shape function based on Lagrange polynomials is applied for the 4-nodes quadrilateral element, and the shape function for constant strain triangles is used for the 3-nodes triangular elements. The Gauss–Legendre quadrature is applied for the numerical integration of the stiffness matrix, which is suitable for linear elastic models.

### 3.1. Mode I Loading with a Horizontal Crack

**Figure 8:**
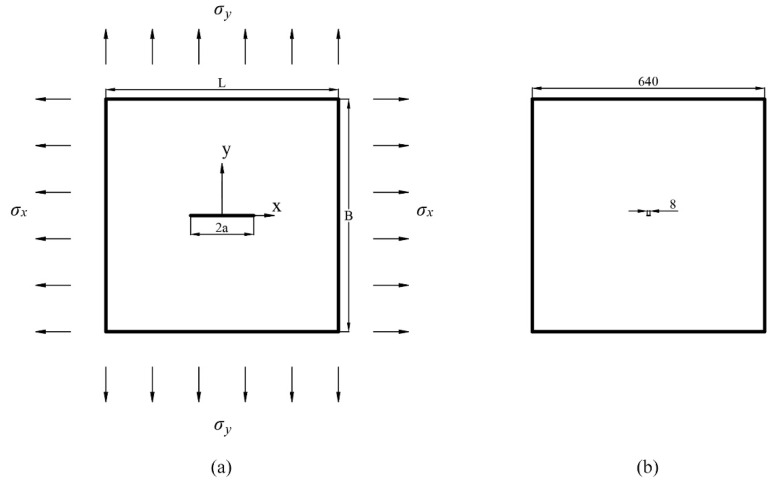
(**a**) Loading condition of the plate with a horizontal crack in the center; (**b**) Size comparison between crack and plate in the example (unit: mm).

In this example, a 640 mm × 640 mm plate with a 4 mm horizontal straight crack in the center is considered. The plate is under biaxial loading; see Figure 8a. More details about the configuration of the simulation are presented in Table 1. Since the boundary length of the plate is 160 times the size of the crack, this example can be regarded as a horizontal crack in a semi-infinite plate under biaxial load; see Figure 8b.

**Figure 9:**
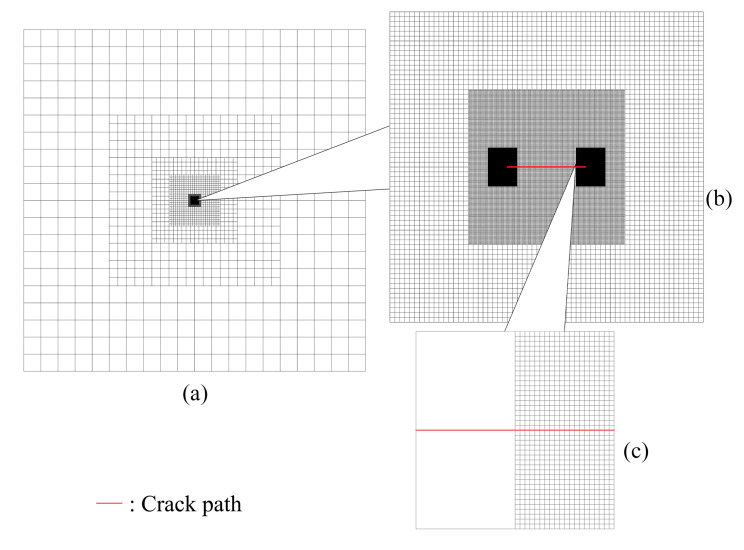
(**a**) Global mesh (in this view, the size of the largest element is 32 mm × 32 mm); (**b**) Mesh from the view of the 5th-level refinement domain (in this view, the size of the largest element is 0.25 mm × 0.25 mm); (**c**) Comparison between the elements from the 7th-level refinement domain and the elements from the 6th-level refinement domain (in this view, the size of the largest element is 0.0625 mm × 0.0625 mm).

**Figure 10:**
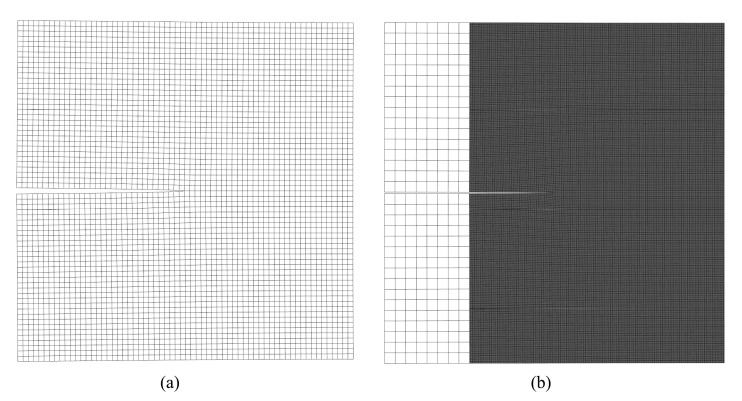
(**a**) Deformed mesh around the crack tip from the view of highest refinement level (in this view, the size of the largest element is 0.003125 mm × 0.003125 mm); (**b**) Deformed mesh around the crack tip from the view of the 6th refinement level (in this view, the size of the largest element is 0.0625 mm × 0.0625 mm).

The mesh refinement is concentrated around the crack tip region see Figure 9, which also includes a magnified presentation of the 5th-level refinement domain, as well as a comparison between the elements from the 7th-level refinement domain and the elements from the 6th-level refinement domain—which shows that the latter is 20 times the size of the former. In this study, since it would be difficult to present the whole mesh density without magnification, the mesh is always shown together with its magnified view. Figure 10 shows the mesh around one of the crack tips after deformation. In this example, if fine mesh is applied to the entire model with the same smallest mesh size, the numerical model will consist of more than 41 billion nodes and elements. Considering the number of nodes and elements presented in Table 1, the application of h-AES method could significantly reduce the computational cost.

In this example, the crack is represented by the edge separation method, since no element split was needed. Compared to the element deletion method, the representation of crack by applying the h-AES method does not remove any elements from the numerical model.

In order to verify the numerical results, they have to be compared to analytical solutions. In this example, Westergaard’s solution and the theory of stress intensity factor (SIF) are used.

**Figure 11:**
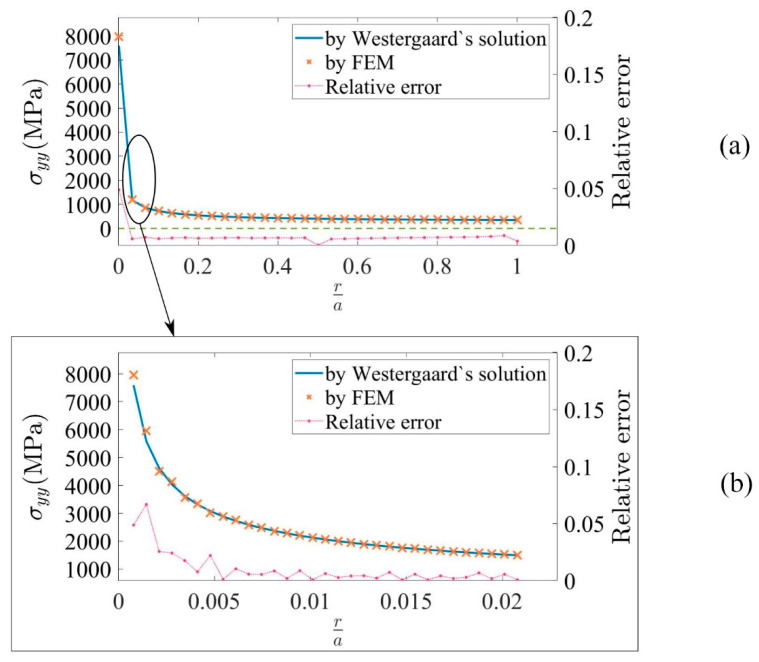
(**a**) Comparison between $\sigma_{yy}$ from the numerical result and Westergaard’s solution when $0<\frac{r}{a}\leq 1$. The relative error in the plot has no unit. (**b**) A magnified view revealing more details of the comparison between $\sigma_{yy}$ from the numerical result and Westergaard’s solution when $0<\frac{r}{a}\leq 0.02$. The relative error in the plot has no unit.

Regarding the coordinate system in Figure 8a, Westergaard’s solution [53] offers a closed-form solution to represent the stress field on y=0. For $\sigma_{yy}$ in the stress field, the function is written as:

(2) $$\sigma_{yy}=\frac{\sigma_{\infty }}{\sqrt{1-{\left(\frac{a}{x}\right)}^{2}}}\;\;$$

where a is half of the length of the crack, x=r+a, r is the distance from crack tip, and $\sigma_{\infty }$ is the far-field stress, i.e., the stress on the boundary of the plate. Figure 11 shows the comparison between the results from h-AES and Westergaard’s solution, with Figure 11b showing more details of the comparison near the crack tip. The shape function used in the program is based on constant strain triangles or Lagrange polynomials, which cannot obtain exact representations of the behavior in the region of singularity [54], which is the crack tip region in this example. As a result, the accuracy near the crack tip is not as good as in other regions. However, when $\frac{r}{a}\geq 0.07$, the relative error between numerical and analytical result is below 1%. In general, a good correspondence is achieved.

In LEFM, the stress field near crack tips can be represented using stress intensity factors (SIF), which are adopted in this study for a comparison between the numerical and the analytical results. In this example, the stress field equation for mode I loading [55] is used:

(3) $$\sigma_{xx}=\frac{K_{I}}{\sqrt{2\pi r}}cos\frac{\theta }{2}\left(1-sin\frac{\theta }{2}sin\frac{3\theta }{2}\right)$$

(4) $$\sigma_{yy}=\frac{K_{I}}{\sqrt{2\pi r}}cos\frac{\theta }{2}\left(1+sin\frac{\theta }{2}sin\frac{3\theta }{2}\right)$$

(5) $$\tau_{xy}=\frac{K_{I}}{\sqrt{2\pi r}}cos\frac{\theta }{2}sin\frac{\theta }{2}cos\frac{3\theta }{2}$$

where $K_{I}$ is the SIF for mode I loading, and r and θ are polar coordinates. For horizontal cracks in an infinite plate under biaxial loading, the value of $K_{I}$ is calculated by the following equation [55]:

(6) $$K_{I}=\sigma_{\infty }\sqrt{\pi a}$$

where $\sigma_{\infty }$ is the far-field stress, i.e., the boundary stress.

**Figure 12:**
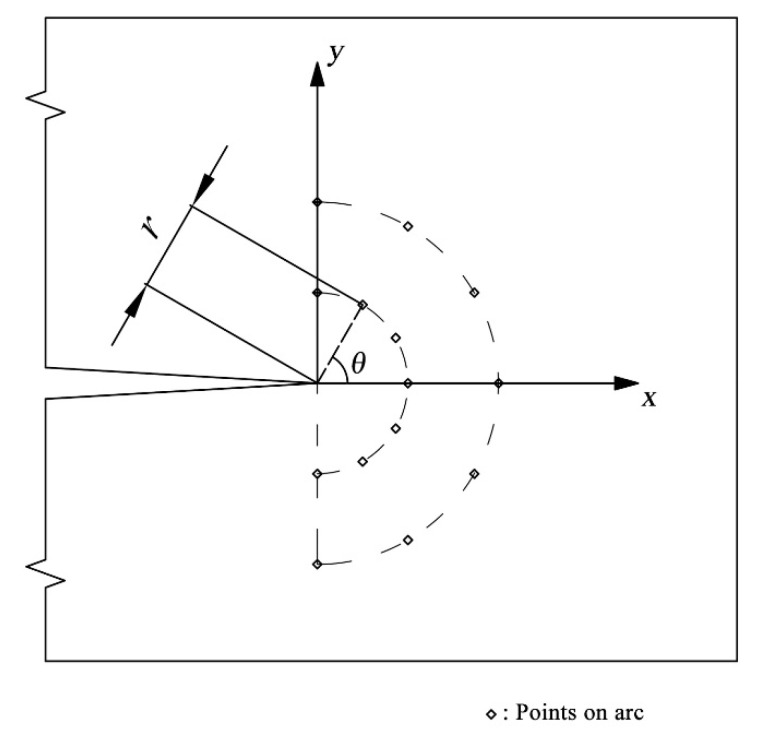
Arc in front of the crack tip for a comparison between stress values from analytical (SIF) and numerical results.

In order to compare numerical and analytical results using the stress field equations, the stress results of different points located on several arcs ahead of the crack tip are selected. Figure 12 shows the configuration of the arc and the points for stress comparison. In this study, three arcs are selected. Each of the arcs has 21 points. The ratio η for the arcs is 0.02, 0.04, and 0.06, respectively, where η=r/a.

**Figure 13:**
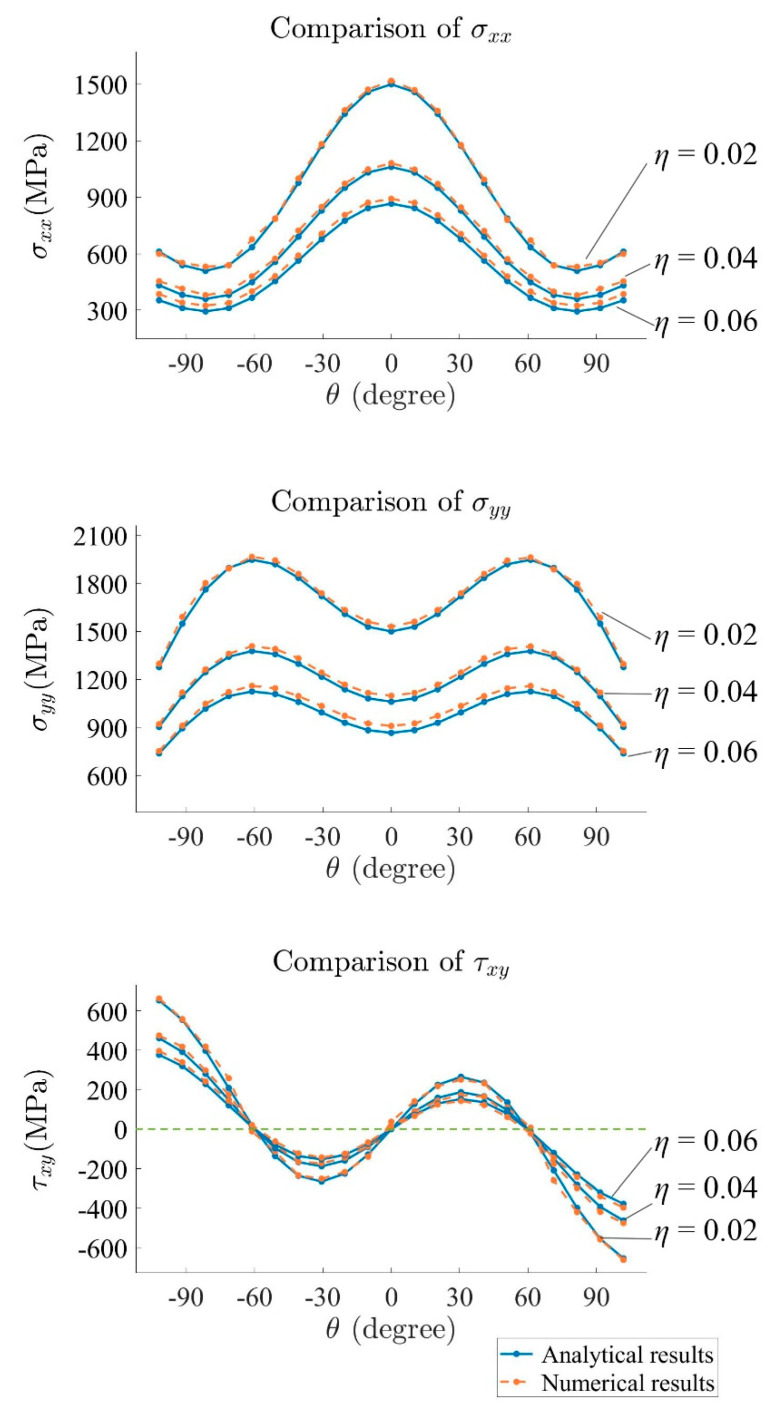
Comparison of stress from numerical and analytical results.

The comparison between numerical and analytical results is shown in Figure 13. For $\sigma_{xx}$,$\;\sigma_{yy}$, and $\tau_{xy}$, the numerical result corresponds best with the analytical value when η=0.02, with the relative error between numerical and analytical result mostly below 5% for $\sigma_{xx}$ and 3% for $\sigma_{yy}$. This is due to the fact that the stress field equation is valid for r→0, which means that, in practice, the accuracy decreases with increasing r. However, for the presented configurations, agreement between closed-form solutions and numerical calculations is very good.

From the equations concerning stress fields around crack tips [55], the following relationship could be deduced: [56].

(7) $${\left(\sigma_{xx}+\sigma_{yy}\right)}_{I+II}=2\left(\frac{K_{I}}{\sqrt{2\pi r}}cos\frac{\theta }{2}-\frac{K_{II}}{\sqrt{2\pi r}}cos\frac{\theta }{2}\right)$$

where $K_{I}$ and $K_{II}$ are the SIF for mode I and II, respectively.

For θ=90° and θ=−90°, $K_{I}$ and $K_{II}$ can be expressed as:

(8) $$K_{I}=\frac{\sqrt{\pi r}}{2}\left({\left({\left(\sigma_{xx}+\sigma_{yy}\right)}_{I+II}\right)}_{\theta =90{}^{\circ}}+{\left({\left(\sigma_{xx}+\sigma_{yy}\right)}_{I+II}\right)}_{\theta =-90{}^{\circ}}\right)$$

(9) $$K_{II}=\frac{\sqrt{\pi r}}{2}\left(-{\left({\left(\sigma_{xx}+\sigma_{yy}\right)}_{I+II}\right)}_{\theta =90{}^{\circ}}+{\left({\left(\sigma_{xx}+\sigma_{yy}\right)}_{I+II}\right)}_{\theta =-90{}^{\circ}}\right)$$

**Figure 14:**
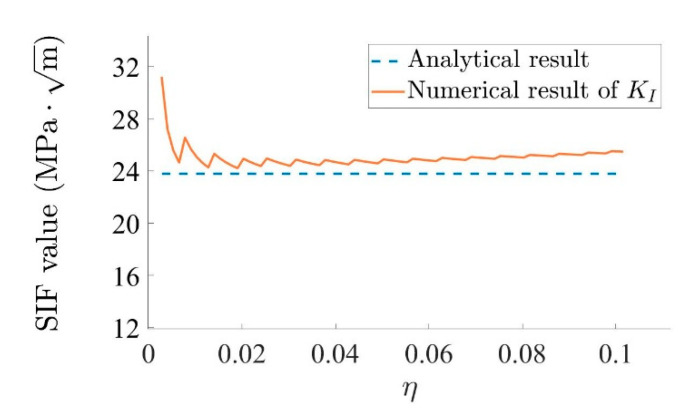
Comparison of numerical and analytical results for $K_{I}$.

By using Equations (7) and (8), the stress intensity factor can be calculated from the stress value, which makes it possible to compare numerical and analytical results for $K_{I}$; see Figure 14. The difference between numerical and analytical results is most pronounced near the crack tip, where singularity exists. This phenomenon also results from the shape functions applied in the program. After η>0.02, when η increases, the difference between numerical results and analytical results increases gradually. This phenomenon can be explained by the fact that the stress field equation is valid for η→0, which is the result of r→0. In general, the numerical result is close to the analytical result.

### 3.2. Mixed-Mode Loading with an Inclined Crack

**Figure 15:**
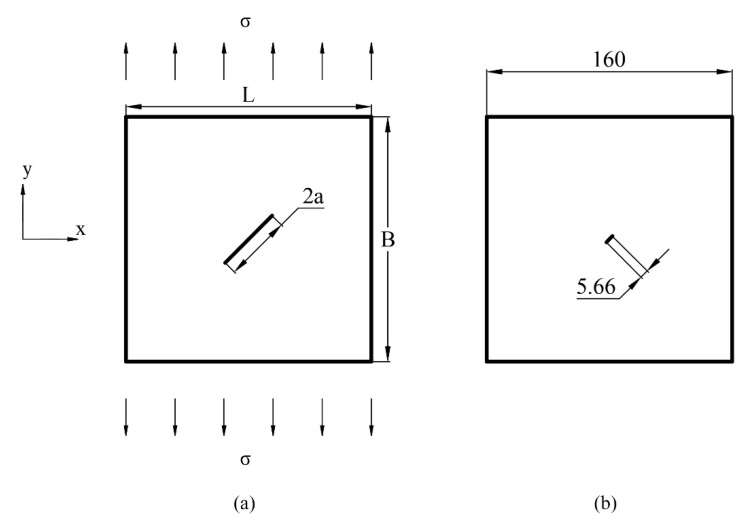
(**a**) Loading condition of the plate with an inclined crack in the center; (**b**) Size comparison between crack and plate in this example.

Considering a square plate under uniaxial loading (see Figure 15) and an inclined crack, the crack is subjected to mode I and mode II loading. In this example, a 160 mm × 160 mm plate under uniaxial loading is considered. The crack in the plate runs straight through the center. The angle from the positive direction of the *x*-axis to the crack is 45 degrees; see Figure 15. More details about the configuration of the simulation are given in Table 2. Since the length of the plate is about 28 times the size of the crack, this example can be regarded as an inclined crack in a semi-infinite plate under uniaxial load; see Figure 15.

**Figure 16:**
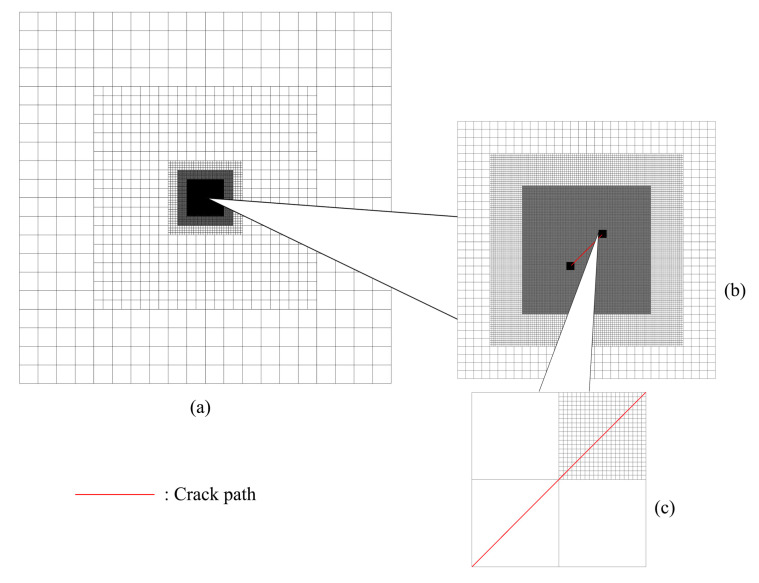
(**a**) Global mesh (in this view, the size of the largest element is 8 mm × 8 mm); (**b**) Mesh from the view of the 2nd-level refinement domain (in this view, the size of the largest element is 1 mm × 1 mm); (**c**) Comparison between the elements from the 5th-level refinement domain with the elements from the 4th-level refinement domain (in this view, the size of the largest element is 0.0625 mm × 0.0625 mm).

**Figure 17:**
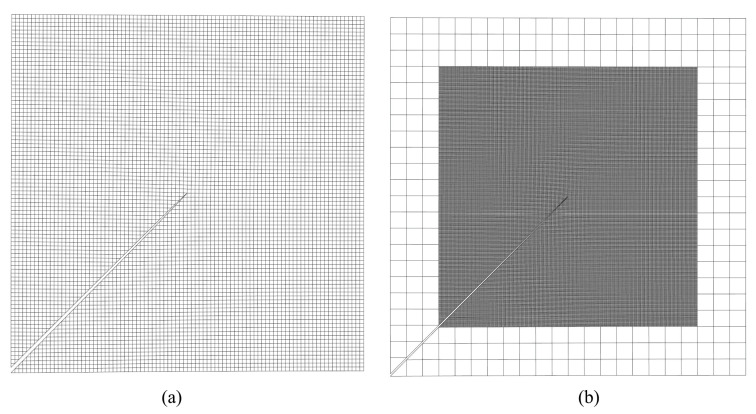
(**a**) Deformed mesh around the crack tip from the view of the highest refinement level; (**b**) Deformed mesh around the crack tip from the view of the 4th refinement level.

The mesh refinement is concentrated around the crack tip. In order to represent the inclined crack, the elements on the crack path are split; see Figure 16c and Figure 17a. Figure 16 shows a magnified view of the 2nd-level refinement domain, as well as a comparison between the elements from the 5th-level refinement domain and the elements from the 4th-level refinement domain, with the latter being 20 times the size of the former. Figure 17 shows the mesh around one of the crack tips after the deformation. In this example, if fine mesh is applied to the entire model with the same smallest mesh size, the numerical model will consist of more than 2 billion nodes and elements. Similar to the last example, considering the number of nodes and elements presented in Table 2, the application of h-AES method could greatly reduce the computational cost.

In this example, the crack is represented by splitting quadrilateral elements into triangular elements and separating the triangular elements along their hypotenuse; see Figure 17. Same as that in the last example, no element was removed from the numerical model to represent the crack as well.

The theory of stress fields using stress intensity factors is used to verify the numerical results. In this example, apart from the stress field equation for mode I loading, the stress field equation for mode II loading [55] is used as well:

(10) $$\sigma_{xx}=\frac{K_{II}}{\sqrt{2\pi r}}sin\frac{\theta }{2}\left(2+cos\frac{\theta }{2}cos\frac{3\theta }{2}\right)$$

(11) $$\sigma_{yy}=\frac{K_{II}}{\sqrt{2\pi r}}sin\frac{\theta }{2}cos\frac{\theta }{2}cos\frac{3\theta }{2}$$

(12) $$\tau_{xy}=\frac{K_{II}}{\sqrt{2\pi r}}cos\frac{\theta }{2}\left(1-sin\frac{\theta }{2}sin\;\frac{3\theta }{2}\right)\;$$

In this example, the $K_{I}$ and $K_{II}$ are [55]:

(13) $$K_{I}=\sigma \sqrt{\pi a}\cdot sin^{2}\theta$$

(14) $$K_{II}=\sigma \sqrt{\pi a}\cdot sin\theta cos\theta$$

**Figure 18:**
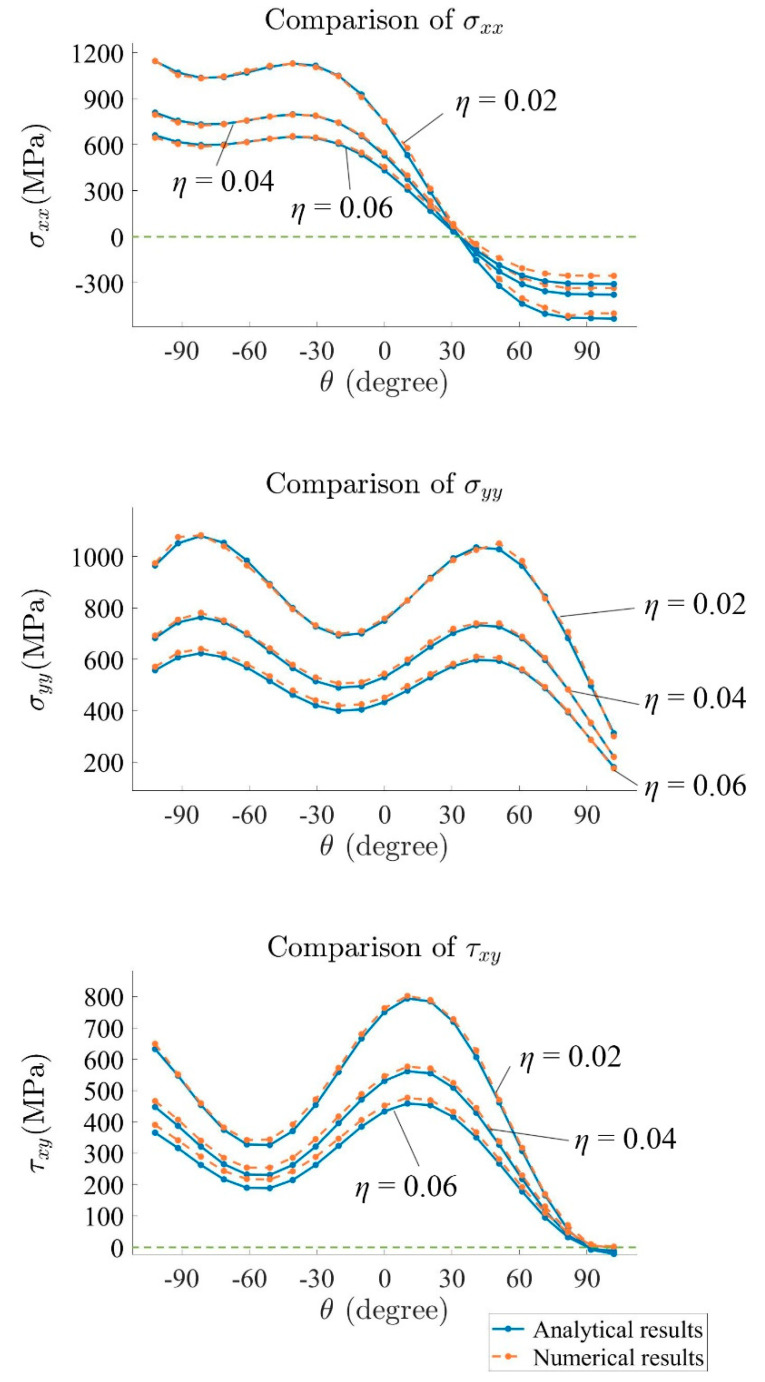
Comparison between stresses from numerical and analytical results.

Similar to the last example (Figure 12), several points in front of the crack tip are chosen for the comparison between the numerical results and analytical results of $\sigma_{xx}$, $\sigma_{yy}$, and $\tau_{xy}$ in Figure 18. Again, the highest accuracy is obtained where η=0.02.

**Figure 19:**
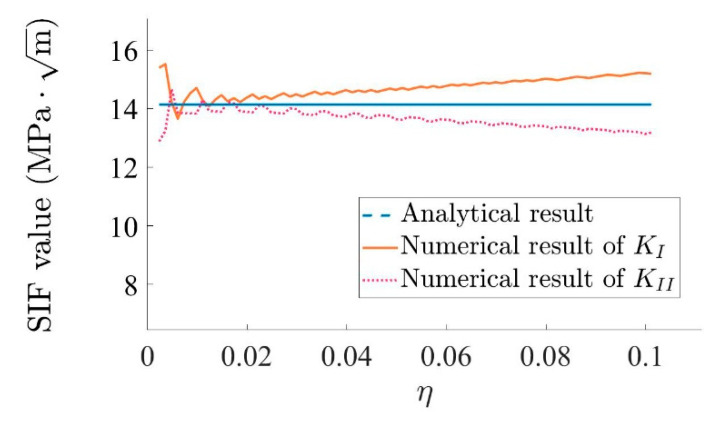
Comparison between numerical and analytical results for $K_{I}$ and $K_{II}$.

Equations (7) and (8) are used to calculate $K_{I}$ and $K_{II}$ from the stress field near the crack tip. Figure 19 shows a comparison between $K_{I}$ and $K_{II}$ from the numerical and analytical results. Due to the fact that θ=45°, it can be concluded from Equations (13) and (14) that the analytical results of $K_{I}$ and $K_{II}$ are the same in this example, as they share the same line in Figure 19. Like in the last example, the difference between the numerical result and the analytical result is the most pronounced near the crack tip. After the position of best accuracy, the difference between numerical result and analytical result increases gradually as η increases. The reason for this is the same as in the last example. Again, the numerical result is close to the analytical result.

## 4. Discussion

In this study, by combining the h-adaptivity and the element splitting method, the h-AES method was introduced for the task of simulating cracks in large structures. The main contributions of this study are summarized as follows:

- The application of h-adaptivity method enables the h-AES method to effectively create very fine meshes while keeping most of the global mesh structured and coarse. Comparing with the application of fine mesh to the entire model, the introduction of h-adaptivity could significantly reduce the computational cost.
- The numerical results of the h-AES method were verified against analytical solutions from LEFM scenarios with good correspondence. As a result, in numerical models mostly consisting of coarse meshes, more local details of FEM-based crack simulations could be revealed by the mesh refinement.
- Considering engineering applications, compared with the frequently applied element deletion method, no element is deleted in the application of the element splitting method. As a result, the drawbacks caused by element deletion, such as the loss of mass and energy, are avoided.
- The element splitting method integrated in the h-AES method is based on the edge separation method, which means that, in the h-AES method, the crack paths still have a strong mesh dependency. However, as the element splitting method is applied, numerical cracks can propagate in the diagonal line of quadrilateral elements, which can provide more flexible crack paths—in particular for structured meshes that initially only included quadrilateral elements. Hence, the extent of the mesh-dependency of crack paths is reduced.

The aim of the h-AES method is to provide a practicable crack representation method for simulations of cracks in large engineering structures under static or cyclic loading. In this study, the h-AES method is applied to linear elastic models with cracks. However, it is possible to apply the h-AES method to more numerical simulation scenarios with additional numerical functions, such as the introduction of non-linear material models and the algorithm of contact problems. Moreover, for ductile materials used in ship structures, the onset of local material failure under critical loading can be captured accurately with coarse meshes employing proper material models [12,13,15,57]. This provides a possibility to adaptively introduce local mesh refinement before crack initiation, which could be included in future research work.

Concerning the examples presented in this study, the increase in computational cost for the mesh refinement is caused by the increased degrees of freedom. If the h-AES method is applied in explicit analysis, since the time step is related to the smallest mesh size, the influence on computational cost will be researched in future study as well.

## 5. Conclusions

In this study, an adaptive mesh refinement method called the ‘h-adaptive element splitting’ (h-AES) method was introduced for the numerical simulation of cracks using shell elements in FEM. Two examples of the h-AES method for crack simulations in large structure under LEFM scenarios were presented. The numerical results were verified against analytical solutions and showed good correspondence. The h-AES method was proven to be able to effectively reveal local details of geometry and material behaviors in numerical models mostly consisting of structured coarse mesh. By employing the h-AES method, very small cracks are well represented in large structures without any deletions of elements. Considering the advantages mentioned above, the implementation of h-adaptivity could benefit the numerical simulation of fatigue or accidental load scenarios involving large structures, such as ship hulls. Future research will integrate more numerical techniques into the h-AES method and apply the h-AES method to more complex simulations. This could be simulations of tensile tests of steel specimens or impact tests on steel panels.

## Figures and Tables

**Table 1:** Configuration of the simulation.

Parameters	Values
Length of the plate (L)	640 mm
Width of the plate (B)	640 mm
Thickness of the plate (t)	1 mm
Crack length (2a)	4 mm
Stress at boundary ($\sigma_{x}$ and $\sigma_{y}$)	300 MPa
Young’s modulus	206 GPa
Mass density	7900 kg/m^3^
Poisson’s Ratio	0.3
Global mesh size	32 mm
Smallest mesh size	0.003125 mm
Refinement levels	7
Number of nodes	636,960
Number of elements	634,036

**Table 2:** Configuration of the simulation.

Parameters	Values
Length of the plate (L)	160 mm
Width of the plate (B)	160 mm
Thickness of the plate (t)	1 mm
Crack length (2a)	5.66 mm
Angle of crack (β)	45°
Stress at boundary (σ)	300 MPa
Young’s modulus	206 GPa
Mass density	7900 kg/m^3^
Poisson’s Ratio	0.3
Global mesh size	8 mm
Smallest mesh size	0.003125 mm
Refinement levels	5
Number of nodes	276,528
Number of elements	279,120

## Nomenclature and Abbreviations

$\Omega_{r}$	The domain of refined mesh.	$\Omega_{r1},\;\Omega_{r2},\;\ldots$	The domains of refined meshes in first, second levels, and so on.
$\Omega_{c}$	The domain of original coarse mesh.		
$l_{e}$	The length of element edge.	$u_{1},\;u_{2},\;\ldots$	The displacements of node 1, node 2, and so on.
a	Half of the crack length.		
L	Length of the plate.	N1, N2, …	Node 1, node 2, and so on.
B	Width of the plate.	$\sigma_{xx},\;\sigma_{yy}$	Normal stress in x and y directions.
t	Thickness of the plate		
r	The distance from crack tip.	$\tau_{xy}$	Shear stress in xy-plane.
$\sigma_{\infty }$	Far-field stress.	$K_{I},\;K_{II}$	Stress intensity factor of mode I, II loading.
θ	The angle of crack in polar coordinates.		
β	The angle of crack on the plate.	η	The ratio of the distance to crack tip and half of the crack length.
FEM	Finite element method		
XFEM	Extended finite element method		
LEFM	Linear elastic fracture mechanics		
h-AES	h-adaptive element splitting		
